# Prospects for Comprehensive Analyses of Circulating Tumor Cells in Tumor Biology

**DOI:** 10.3390/cancers12051135

**Published:** 2020-05-01

**Authors:** Masahiko Aoki, Hirokazu Shoji, Ayumi Kashiro, Keiko Takeuchi, Yoshihiro Shimizu, Kazufumi Honda

**Affiliations:** 1 Department of Biomarkers for Early Detection of Cancer, National Cancer Center Research Institute, Tokyo 104-0045, Japan; aokidayo110@gmail.com (M.A.); akashiro@ncc.go.jp (A.K.); ketakeuc@ncc.go.jp (K.T.); 2 Gastrointestinal Medical Oncology Division, National Cancer Center Hospital, Tokyo 104-0045, Japan; hshouji@ncc.go.jp; 3 Internal Medicine (II), Osaka Medical College, Osaka 569-8686, Japan; 4 RIKEN Center for Biosystems Dynamics research (BDR), Osaka 565-0874, Japan; yshimizu@riken.jp

**Keywords:** circulating tumor cells (CTCs), circulating tumor dna (ctDNA), genomics, metabolomics, heterogeneity, single cell

## Abstract

The comprehensive analysis of biological and clinical aspects of circulating tumor cells (CTCs) has attracted interest as a means of enabling non-invasive, real-time monitoring of cancer patients and enhancing our fundamental understanding of tumor metastasis. However, CTC populations are extremely small when compared to other cell populations in the blood, limiting our comprehension of CTC biology and their clinical utility. Recently developed proteomic and genomic techniques that require only a small amount of sample have attracted much interest and expanded the potential utility of CTCs. Cancer heterogeneity, including specific mutations, greatly impacts disease diagnosis and the choice of available therapeutic strategies. The CTC population consists primarily of cancer stem cells, and CTC subpopulations are thought to undergo epithelial–mesenchymal transition during dissemination. To better characterize tumor cell populations, we demonstrated that changes in genomic profiles identified via next-generation sequencing of liquid biopsy samples could be expanded upon to increase sensitivity without decreasing specificity by using a combination of assays with CTCs and circulating tumor DNA. To enhance our understanding of CTC biology, we developed a metabolome analysis method applicable to single CTCs. Here, we review―omics studies related to CTC analysis and discuss various clinical and biological issues related to CTCs.

## 1. Introduction

Cancer is the second leading cause of death in the United States [1]. Comprehensive analysis of the biological and clinical aspects of circulating tumor cells (CTCs) has attracted considerable interest as a means of enabling the non-invasive, real-time monitoring of cancer patients and enhancing our fundamental understanding of tumor metastasis. CTCs, which are released into the bloodstream from primary tumors and metastatic lesions, are now used as liquid biopsy markers that reflect the biological and clinical aspects of cancers, thus facilitating non-invasive, real-time monitoring of cancer patients. Since CTCs were first observed by Thomas Ashworth in 1869 [2] and analysis of CTCs as “liquid biopsy” was first mentioned by Pantel and Alix-Panabieres in 2010 [3], many scientists have demonstrated the usefulness of CTCs as liquid biopsy prognostic biomarkers [4,5,6,7] and for evaluating the efficacy of treatments for a variety of cancers [8,9,10], even in the early stages of disease [11].

A drawback to the use of CTCs is their rarity. The median CTC count in various cancers is only 1 to 84 per 7.5 mL of peripheral blood [12]. Indeed, only 1.43% of patients with progressive breast cancer have more than 500 CTCs per 7.5 mL of blood [13]. Although the CTC population is extremely small when compared to other cell populations in the blood, thus limiting our understanding of their biology and clinical utility, unlike the case of tissue biopsy repeated sampling to obtain more cells is possible. This makes CTCs an ideal and clinically practical material for investigations of not only basic biological and clinical characteristics of cancer cells but tumor heterogeneity and drug resistance as well [14]. Due to recent technological advances, proteomic and genomic techniques that require only a small amount of sample are now available. These techniques have attracted considerable interest and expanded the potential utility of CTCs. Here, we review―omics studies related to CTC analysis and discuss various clinical and biological issues related to CTCs.

## 2. Cancer Heterogeneity

The heterogeneity of cancers, including specific mutations, greatly impacts disease diagnosis and the choice of available therapeutic strategies. The advent of genomic medicine and single-cell analysis techniques has increased research focused on intratumoral heterogeneity in individual patients. Intratumoral heterogeneity includes the uneven distribution of genetically diverse tumor subpopulations across different lesions as well as dynamic variations in the genetic diversity of individual tumors over time [15]. Substantial intratumoral heterogeneity was revealed by analyses of different regions of 327 tumors independently sampled from 100 patients with early stage non-small cell lung cancer (NSCLC) [16]. Similar intratumoral heterogeneity has been reported for other cancer types [17,18,19]. Johnson et al. reported that the degree of intratumoral heterogeneity is highly variable; in patients with glioma, they found that the number of coding mutations varied from 0 to over 8000 within primary tumors or between primary and metastatic or recurrent sites [20]. In a study of six lung cancer patients with MET amplification and the epidermal growth factor receptor (EGFR) T790M mutation, 33 gefitinib-refractory lesions were collected at autopsy [21]. Each patient harbored identical activating mutations in the EGFR gene within their tumors. In two of the six patients, the T790M mutation and/or MET amplification was present, depending on the lesion site. In contrast, four other patients had either the T790M mutation or MET amplification in all metastatic sites. A mutual complementary relationship between the incidence of the T790M mutation and the degree of MET amplification was observed in these gefitinib-refractory tumors.

The evidence clearly indicates that tumors exhibit heterogeneity and may differ before and after cancer treatment. This heterogeneity can lead to different responses to therapy. Single CTCs have been used to elucidate the complex tumor genomic profiles of colon cancer tumors [22]. Mutations detected within driver genes (e.g., adenomatous polyposis coli (APC), KRAS, or phosphatidylinositol-4,5-bisphosphate 3-kinase catalytic subunit alpha (PIK3CA)) in primary tumors and metastatic sites were also found in the corresponding CTCs. In some cases, however, mutations have only been identified in CTCs. For example, one patient harbored 25 mutations in 17 genes in the analyzed samples. Among these mutations, four constitutional mutations were detected in all of the samples. In addition, three somatic mutations were identified in all of the tumor samples, including the CTCs. Two other mutations were detected in metastatic sites and CTCs. The rest of the mutations were identified in single CTCs. Most driver mutations associated with cancer development that were first detected only in CTCs were also present at the subclonal level in the primary lesions and metastatic sites of this patient. Even though a correlation was found between mutations in driver genes and single CTCs, single-cell analysis revealed the presence of different mutation patterns in single CTCs from primary and metastatic sites. This study highlighted the possibility that CTCs could be used as liquid biopsy specimens, thus providing a more effective strategy for monitoring genomic profiles that are prone to change during cancer progression, treatment, and relapse, at the primary site of a tumor. This study also revealed that the mutation profiles of single CTCs do not perfectly match the mutation profiles of primary tumors or metastatic sites. Collectively, these results suggest that CTCs exhibit marked heterogeneity at the subclonal level.

## 3. Epithelial-Mesenchymal Transition

Traditionally, CTCs are thought to induce epithelial–mesenchymal transition (EMT) during dissemination. Although normal epithelial cells are immobile, some cancerous epithelial cells may begin expressing proteins associated with motility and cease expressing proteins involved in forming connections between cells during embryonic development. This transformation produces mobile ‘mesenchymal’ cells that can migrate and form other lesions. Cancer cells that have an EMT phenotype can penetrate and pass through blood vessel walls and enter the bloodstream. These CTCs eventually attach to endothelial cells of blood vessels and exit the bloodstream, forming new metastatic lesions in other organs [23,24,25]. Three types of CTCs have been described: epithelial, mesenchymal, and epithelial/mesenchymal hybrids [26].

Moreover, EMT is associated with the properties of cancer stem cells (CSCs). The metastatic potential of a tumor depends upon the appearance of CSCs in the primary tumor tissue. These CSCs exhibit two characteristics: self-renewal and the ability to efficiently regenerate phenotypic heterogeneity in the parental tumor [27,28,29,30]. Therefore, the induction of EMT leads to the expression of stem cell markers, increased self-renewal, and increased tumor-initiating potential [31,32]. However, which CTC subtype exhibits the highest metastasis-initiating activity remains unclear, but it was recently reported that epithelial-type CTCs have a higher potential to translate protein and proliferate [33]. From another report, though EMT led to improve capacity to migrate, epithelial-type CTCs had the most metastatic potential and the proportion of epithelial-type CTCs had associations with distant metastases and prognosis [34]. Hence, immunoaffinity-based enrichment technologies relying on epithelial cell surface markers can underestimate the number of CTCs, but may quantify metastatic potential cells.

## 4. CTC Enrichment

Various CTC enrichment technologies have been developed [35], including density gradient centrifugation [36,37,38], microfiltration in two and three dimensions [36,39,40,41], inertial microfluidics [42,43], dielectrophoresis (DEP) [44], acoustophoresis [42], direct imaging modalities [42,43,44,45], functional assays [42], and immunoaffinity techniques [46,47,48,49] (Table 1). Each of these technologies has specific working principles and different features. Density gradient centrifugation is separation based on the migration of cells through a medium of varying density. Microfiltration in two and three dimensions involves the filtration of a sample through an array of microscale constrictions and isolation of CTCs based on size and deformability. Inertial microfluidics is a size-dependent separation technique based on the position of cells in a flow channel. DEP separates cells based on their electrical properties as they pass through a non-uniform alternating current field. Acoustophoresis is a size-dependent separation method based on the acoustophoretic mobility of cells. Direct imaging modalities integrate microscopy and flow cytometry for the identification of specific subpopulations of cells. Functional assays enable enrichment of target cells based on the bioactivity of viable cells. Immunoaffinity technologies enable positive or negative cell enrichment based on selected antibodies and target antigens. Aneuploidy (the aberrant alternation of chromosomes) occurs in cancer cells, and aneuploidy of chromosomes in CTCs exhibiting drug resistance have been reported [50]. To detect aneuploid CTCs, immunostaining-fluorescence in situ hybridization (iFISH) is applied. Among the technologies for CTC enrichment, the only one approved by the U.S. Food and Drug Administration for use in clinical settings is the CellSearch system. However, immunoaffinity-based enrichment technologies relying on epithelial cell surface markers (e.g., epithelial cell adhesion molecule (EpCAM) or cytokeratins (CKs)) can miss CTCs that have undergone EMT or those that exhibit stem cell potential, which could lead to underestimation of the total number of actual CTCs present in the bloodstream. In addition, patients with benign disease typically have a lower frequency of EpCAM- and CK-positive CTCs when compared to patients with malignant cancer [51]. To overcome these drawbacks, development of label-free CTC enrichment technologies has received considerable research attention.

Microfluidic technologies are well suited for label-free analyses. As such, microfluidic methods for size-based isolation and concentration of cells (positive test rate using cell line of 84%) as well as automated size-based selection (positive test rate using cell line between 42% and 70%) have been developed [52,53]. In addition, Hou et al. (2013), reported a novel microfluidic flow method for isolating CTCs [43]. Their method, based on the application of inertial microfluidics, enriches intact and viable CTCs. The sensitivity and specificity of the microfluidic flow method for detecting CTCs were 80.4% and 85.7%, respectively, based on analyses of 77 clinical samples from 21 healthy donors and 56 cancer patients [54,55]. This system efficiently isolates CTCs without the need for affinity purification using antibodies against epithelial biomarkers, thus avoiding potential underestimation of CTC subpopulations undergoing EMT, such as CTCs exhibiting downregulated expression of EpCAM. In addition, because this system does not require antibodies, which could produce unwanted biological effects, it is advantageous for isolating CTCs for subsequent culture or biological analyses, such as proteomic or metabolomic studies.

## 5. Increasing Sensitivity for the Mutation Profile Using a Combination of Assays for CTCs and Circulating Tumor DNA

The sensitivity of genomic profiling using liquid biopsy specimens could be increased without decreasing specificity by employing a combination of assays for CTCs, circulating tumor DNA (ctDNA), and cell-free DNA (cfDNA).

Table 2 lists previous reports describing the use of combinations of assays for CTCs, ctDNA, and cfDNA. Combinations of assays for determining the number of CTCs and analyzing ctDNA and cfDNA have been reported for breast cancer [56,57], follicular lymphoma [58], non-small cell lung cancer (NSCLC) [59,60], and urothelial cancer [61]. In breast cancer, the number of CTCs detected, the ratio of the number of mutant genes to the total number of genes at a given genomic position (%ctDNA), the number of alterations, and levels of cfDNA were associated with prognosis. In follicular lymphoma, a significant correlation was found between the total tumor volume and both the number of CTCs detected and cfDNA level. In NSCLC, the EGFR mutation status of cfDNA was analyzed. The presence of EGFR mutations detected in CTCs was correlated with the presence of cfDNA. Another study of patients who underwent immune checkpoint therapy with nivolumab showed that those patients in which the baseline cfDNA level and number of CTCs were below the median survived significantly longer than patients in which the baseline number of CTCs and cfDNA level were above the median values. In urothelial cancer, no correlation was observed between the number of CTCs and ctDNA. A ctDNA fraction >2% was associated with significantly worse prognosis compared with patients in which CTCs could not be detected.

**Figure 1:**
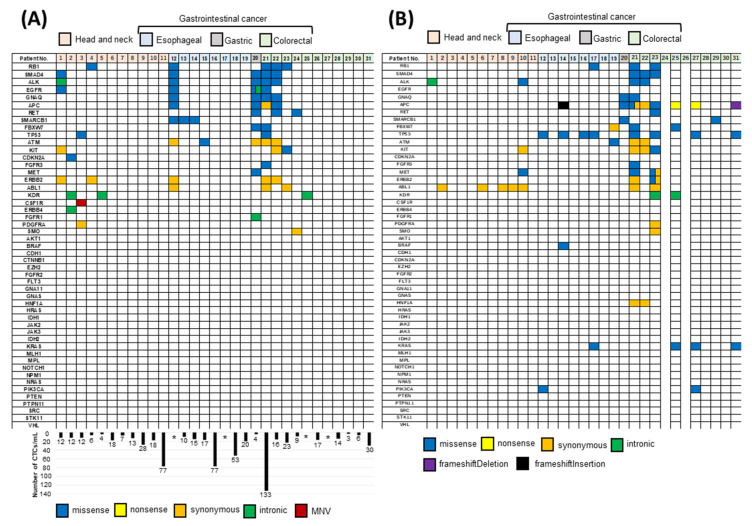
Combined analysis of genomic alterations in circulating tumor cells (CTCs) and circulating tumor DNA (ctDNA) using targeted next-generation sequencing. (**A**) Genomic alterations in CTCs of head and neck cancer, esophageal cancer, gastric cancer, and colorectal cancer patients. The number of CTCs is indicated in the columns. * The number of CTCs could not be determined in 4 patients. (**B**) Genomic alterations in ctDNA from patients with head and neck cancer, gastric cancer, and colorectal cancer. ctDNA could not be extracted from 2 patients with colorectal cancer. Blue, yellow, orange, green, purple, and black spaces represent missense mutations, nonsense mutations, synonymous mutations, intronic mutations, frameshift deletions, and frameshift insertions, respectively [62].

Recently, we investigated whether CTCs can be used as a tool for selecting clinical strategies by observing molecular changes in real time in patients with advanced stages of cancer [62]. We enriched CTCs from four different cancer types (head and neck, esophageal, gastric, and colorectal) using the microfluidics flow method and carried out targeted sequencing of CTCs and ctDNA using next-generation sequencing (NGS). We designed an experimental strategy that combined analyses of alterations in the genomic profiles of CTCs and ctDNA from individual patients who underwent therapy with anti-EGFR antibodies. Using this approach, we identified unique mutations in the CTCs and ctDNA of individual patients with metastatic colorectal cancer before and after anti-EGFR therapy. However, concordance was not always observed between the genetic mutation profiles of the CTCs and ctDNA of individual patients (Figure 1A,B), and the concordance rate was generally low. These results suggest that the genetic alteration profiles of the CTCs and ctDNA differed. Therefore, combined analyses of CTCs and ctDNA could improve the detection of genomic alterations when compared to assays targeting CTCs or ctDNA alone. We identified missense mutations in 5 out of 10 cases of head and neck cancer (50%) and 15 out of 18 cases of gastrointestinal cancer (83.3%) using this combination analysis approach. The same amino acid changes were detected in both CTCs and ctDNA in 6 of the 28 total cases. Our data indicate that CTCs and ctDNA exhibit genetic heterogeneity, such that both must be evaluated for optimal monitoring of disease progression and treatment selection in the clinical setting. Indeed, we identified increased rates of mutation in the *KRAS*, *NRAS*, and *PIK3CA* genes in patients exhibiting resistance to anti-EGFR therapy via combined NGS analysis of CTCs and ctDNA. Moreover, mutations in codon 61 in *KRAS* and *NRAS* were detected more frequently in colorectal cancer patients with acquired resistance to anti-EGFR therapy than before initiation of anti-EGFR therapy.

In another study of 28 patients with multiple myeloma [63], discordance was observed in the tumor fractions of enriched CTCs and cfDNA. A higher tumor fraction was detected in cfDNA compared with enriched CTCs in several patients, but there were also patients in which the tumor fraction was higher in enriched CTCs. For example, one patient had a tumor fraction of 91% in cfDNA and 4% in the enriched CTCs, whereas another patient had a tumor fraction of 80% in the enriched CTCs and 6.7% in ctDNA. As a result, there was no correlation between the tumor fractions of cfDNA and enriched CTCs in the 28 samples examined. These data suggest that CTCs and ctDNA have different genetic alteration profiles. Therefore, combining analyses of CTCs, ctDNA, and cfDNA could enable more sensitive detection of genetic alterations without decreasing the specificity, thus facilitating the establishment of precision oncology.

In our recent study, we used the microfluidics flow method to enrich CTCs and found an average of 14.5 CTCs/mL of blood (range, 3 to 133 CTCs/mL) in one patient, and CTCs were observed in 27 of 31 patients enrolled in our study [62]. These results suggest that the label-free microfluidics flow method enables more efficient enrichment of CTCs that have undergone EMT compared with immunoaffinity-based enrichment technologies.

## 6. Metabolome Analysis With a Single CTC

**Figure 2:**
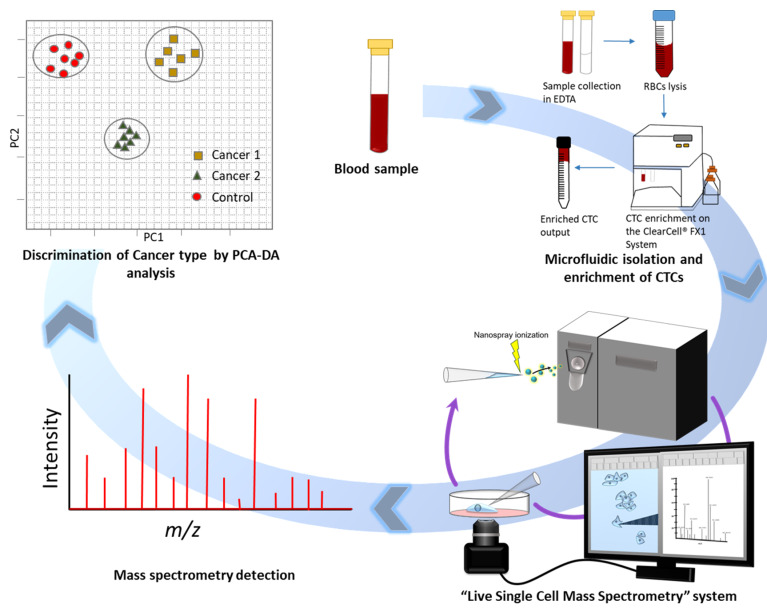
Schematic illustration of the live single-cell mass spectrometry (LSC-MS) method. Blood samples were collected from patients with gastric cancer and colorectal cancer. A microfluidics technique was used to enrich circulating tumor cells (CTCs). Single CTCs were sampled and analyzed using the LSC-MS system [64]. PCA-DA, principal component analysis–discriminant analysis; EDTA, ethylenediaminetetraacetic acid; RBC, red blood cell.

**Figure 3:**
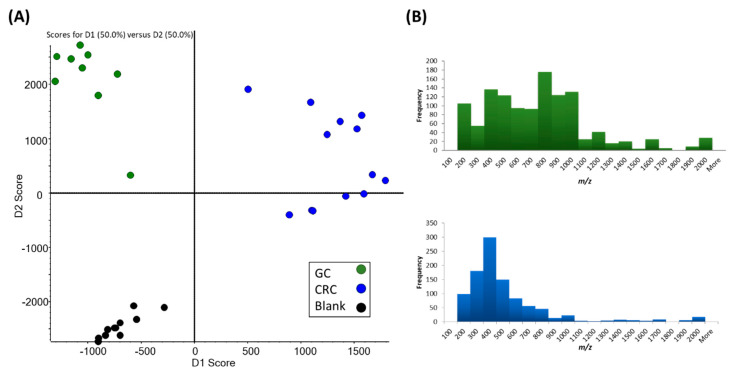
Profiling of gastric cancer (GC) and colorectal cancer (CRC) circulating tumor cells (CTCs) at the single-cell level. (**A**) Principle component analysis–discriminant analysis to distinguish GC CTCs, CRC CTCs, and blank cells. Each dot represents a single cell. (**B**) Histogram of the frequency of peak distribution across the *m/z* scale for GC and CRC [64].

To enhance our understanding of CTC biology, we developed a metabolomic analysis method that can be performed with a single CTC [64]. Although unique metabolomic profiles in the primary tumor site have been reported for different cancer types [65,66,67], we were the first to report the metabolomic profiles of single CTCs from gastrointestinal cancer. In this study, by integrating live single-cell mass spectrometry (LSC-MS) and a microfluidics-based CTC enrichment technique, untargeted analysis was undertaken for CTCs obtained from patients with gastric and colorectal cancer (Figure 2). For LSC-MS, a single cell is captured in a tapered glass microcapillary under video microscopy, and then the cell is ionized and directly inserted into the mass spectrometer. This technique has also been applied to other types of cells [68,69]. In this study, we investigated whether CTCs and lymphocytes obtained from different patients could be distinguished at the single-cell level and whether we could distinguish CTCs obtained from different cancer types. As shown in Figure 3A, even though samples obtained from different patients exhibited different profiles, the CTCs clustered into two distinct groups corresponding to the original cancer type. This suggests that CTC metabolomic characterization could become an efficient tool for cancer diagnosis in the future. By further analyzing the data obtained from gastric cancer samples, in which a high *m/z* peak was detected, we identified a trend in the frequency of peaks distributed along the *m/z* scale. This trend was noticed following the comparison of histograms of the average spectra of CTCs from gastric cancer patients to those from colorectal cancer patients, as shown in Figure 3B. As primary metabolites have a relatively low molecular weight, the increased number of peaks of relatively high molecular weight in the CTCs of gastric cancer patients suggested a distinctive metabolic hallmark for this cancer that most likely involves a higher distribution of lipids, which could be a potential future biomarker for gastric cancer. Furthermore, we analyzed gastric cancer and colorectal cancer patient groups (*n* = 9 and *n* = 13, respectively) to identify possible metabolites or lipids that are unique to these specific cancers. Among the statistically significant peaks identified, acyl carnitine metabolites and sterol lipids levels were more elevated in colorectal cancer than gastric cancer. Eicosanoids were also more abundant in the CTCs of colorectal cancer patients, a finding that was corroborated by other studies examining this cancer type [70].

Although robust analysis of MS signals from single cells remains a challenging task, our method has the potential to be utilized as a novel biomarker on the single-cell level.

## 7. Culture of CTCs

Culturing CTCs represents a future challenge and goal. Several reports of attempts to culture CTCs have been published. CTC culture is very challenging due to the extremely low number of CTCs when compared to other cell populations in the blood and because the behavior of CTCs is unclear. The formation of heterotypic cell clusters between CTCs and white blood cells (WBCs) has been previously reported [71,72,73], and this interplay between CTCs and WBCs has been linked to a worse prognosis [74,75]. A recent study involving both patients and a mouse model revealed that in the majority of cases, these WBCs are neutrophils [76]. The formation of clusters between CTCs and neutrophils is caused by vascular cell adhesion molecule 1, which is involved in the formation of cell-to-cell adhesions [73]. In addition, neutrophils mediate the formation of neutrophil extracellular traps (NETs) during infection. NETs can capture CTCs at sites distant to the primary lesion and thus promote metastasis [77]. Therefore, various methods, such as those that enable CTC enrichment or clustering with WBCs, have been attempted for the culture of CTCs.

To date, there are reports of successful culture of CTCs from colon cancer [9], head and neck cancer [78], and breast cancer [79] by employing CTC enrichment methods. Successful culture of breast cancer CTCs using a method that involves clustering with WBCs was also reported [80]. Such reports of successful culturing of CTCs remain few, however, and although the culturing of CTCs is still challenging, it may lead to the development of new omics (genomic, transcriptomic, proteomic, metabolomic, and secretomic) analytical methods that can further enhance our understanding of CTC biology. The development of CTC culturing techniques may also facilitate new drug screening methods, and there are also approaches for evaluating patient drug responses using an integrated microfluidic system involving microfabricated microwells [81]. Integrating custom microfabricated tapered microwells with microfluidics allows for the formation of robust CTC clusters without the need for pre-enrichment as well as subsequent drug screening in situ. Feedback can be obtained rapidly (after only 2 weeks), thus enabling immediate intervention upon detection of drug resistance or tolerance. This new technique could potentially be used to evaluate patient prognosis during treatment using CTC clusters.

## 8. Conclusions and Perspectives

**Figure 4:**
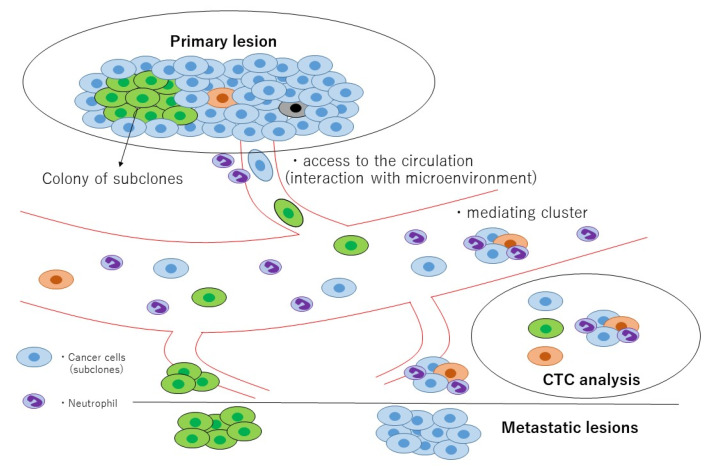
Graphical overview of tumor heterogeneity and circulating tumor cell (CTC) analysis. Heterogeneity is caused by (1) subclones present within the primary lesion, (2) selected cancer cells shed from the primary lesion that invade the blood vessels (e.g., interaction with the microenvironment surrounding tumor). CTC analysis is a useful tool for characterizing this heterogeneity.

The heterogeneity of cancers, including specific mutations, greatly impacts disease diagnosis and the choice of available therapeutic strategies. Tumors are heterogeneous, and they may also exhibit differences based on the time of assessment (e.g., before versus after cancer treatment). Heterogeneity is also related to differences in response to therapy. As noted above, cancer heterogeneity may lead to discordance between the results of assays for primary and metastatic lesions, as well as for CTCs, ctDNA, and cfDNA. A number of studies on the biological behavior of tumor cells have been reported. Rapid autopsies of seven individuals who died of advanced pancreatic cancer were reported [82]. To determine whether clonal evolution occurred within the primary cancer lesion or within secondary sites, the primary tumors were divided into numerous sections, organized three-dimensionally, and then examined. Analyses of distinct regions of the primary tumors clearly indicated the presence of subclones that gave rise to each of the metastases. Additionally, the sizes of the regions indicated that these subclones were not small. The subclones were placed into an ordered hierarchy, establishing an evolutionary path for tumor progression, and the genetic heterogeneity of the metastases reflected the heterogeneity already present within the primary carcinomas. From another view, tumor dissemination can also cause cancer heterogeneity. Cancer cells shed from the primary lesion that invade the blood vessels may be selected [83] (Figure 4).

These results suggest that there is slight concordance between the genetic alteration profiles of primary/metastatic sites and CTCs. Moreover, the biological behavior of tumor cells can change on a moment-to-moment basis, and clonal evolution can occur in primary tumors or in response to selective pressures associated with cancer therapies. We should not focus only on concordance between the primary tumor site and CTCs as a surrogate biomarker for monitoring the primary sites; instead, we should focus on the clinical association between CTCs and clinical findings, such as metastatic potential, the efficacy of targeted therapies, and long-term prognosis.

Liquid biopsy techniques allow for repeated sampling, which is not usually possible with tissue biopsies. Because they are cancer cells, CTCs could be used to determine the biological characteristics of cancers in individual patients. Indeed, melanoma patients with detectable programmed cell-death ligand 1 (PD-L1)-positive CTCs exhibited significantly longer progression-free survival (PFS) following treatment with an immune checkpoint inhibitor than patients with PD-L1-negative CTCs (26.6 months vs. 5.5 months, respectively) [84]. The 12-month PFS rates were 76% and 22% in the PD-L1-positive and PD-L1-negative CTC groups, respectively. In a study of NSCLC patients, upon disease progression, all patients exhibited an increase in PD-L1-positive CTCs, whereas no change or a decrease in PD-L1-positive CTCs was observed in the responding patients [85]. Representative clinical trials involving CTCs are summarized in Table 3 [86].

Subpopulations of CTCs are thought to undergo EMT during dissemination, and as such, the CTC population consists primarily of CSCs. CSCs are associated with both initiation of primary tumors and the establishment of metastatic lesions. EMT and the acquisition of stem cell properties are related (i.e., induction of EMT leads to the expression of stem cell markers, increased self-renewal, and increased tumor-initiating potential). A recent study reported the use of CD44, CD133, and vimentin as expression markers [87]. It is thus important to elucidate the biological characteristics of CTCs. To enhance our understanding of CTC biology, a metabolomic method that can be performed with a single CTC was developed. Each cancer type has a specific metabolic profile, and metabolomic analysis methods could therefore be useful for revealing the biological behavior of CTCs as well as investigating potential new biomarkers on the single-cell level.

The development of novel CTC culturing technologies may enable the evaluation of drug response by taking advantage of liquid biopsy, a minimally invasive real-time monitoring approach, thus guiding drug discovery and development as well as therapeutic decision-making for personalized treatment.

## 9. Summary

Various CTC enrichment technologies have been developed, including density gradient centrifugation, microfiltration in two and three dimensions, inertial microfluidics, DEP, acoustophoresis, direct imaging modalities, functional assays, and immunoaffinity techniques. Therefore, CTCs can now be used as a liquid biopsy marker that reflects the biological and clinical aspects of cancers and enables the non-invasive real-time monitoring of cancer patients. Tumors are heterogeneous and may differ before and after cancer treatment. This heterogeneity can lead to different responses to therapy. Subpopulations of CTCs are thought to undergo EMT during dissemination, and as such, the CTC population consists primarily of CSCs. To better understand tumor cell populations, we demonstrated that information regarding alterations in genomic profiles as determined by NGS analysis of liquid biopsy samples could be expanded upon by employing a combination of assays involving CTCs and ctDNA, thus increasing sensitivity without decreasing specificity. Moreover, to enhance our understanding of CTC biology, we developed a metabolomic analysis method that can be performed with a single CTC. Each cancer type has a specific metabolic profile that could provide new biomarkers that could be monitored on the single-cell level. Among future challenges and goals is the culturing of CTCs. The development of techniques for culturing CTCs may lead to the development of novel omics (genomic, transcriptomic, proteomic, metabolomic, and secretomic) analyses and new drug screening techniques.

## Figures and Tables

**Table 1:** Technologies for circulating tumor cell (CTC) enrichment.

Method	Principle of Technology	Feature
Inertial microfluidics	Position of cells in a flow channel. Size-dependent separation.	Label-free. Enrich intact and viable cells, but false-negatives due to size.
Immunoaffinity techniques	Antibodies and target antigens. Positive (CTC) or negative (other blood cells) selection.	Relatively high sensitivity, but false-negatives due to epithelial–mesenchymal transition.
Density gradient centrifugation	Migration of cells through a medium of varying density.	Label-free. Low cost, but low purity.
Microfiltration in two and three dimensions	Filtration of a sample through an array of microscale constrictions. Size- and deformation-dependent separation.	Label-free. Low cost, but low purity and false negatives due to size variations.
Dielectrophoresis	Electrical properties of target cells as they pass through a non-uniform alternating current field.	Enables capture of single cells, but pre-enrichment is required.
Acoustophoresis	Acoustophoretic mobility of cells. Size-dependent separation.	High cell viability. Recovery efficiency dependent on blood concentration.
Direct imaging modalities	Identification of specific subpopulations of cells using microscopy and flow cytometry.	Real-time fluorescence intensity. Time consuming.
Functional assays	Bioactivity of viable cells. Enrichment of target cells.	High sensitivity, but time consuming and requires continuous activity.

**Table 2:** Summary of combination assays for circulating tumor cells (CTCs), circulating tumor DNA (ctDNA), and cell-free DNA (cfDNA).

Cancer Type	CTC Enrichment	Number of Patients and Sensitivity	Average Number of CTCs (range)	CTC Analysis Method	ctDNA and cfDNA Analysis Method	Authors
Breast cancer	Immunoaffinity (EpCAM, CKs)	91 Not provided	2 CTCs/sample in all patients (0–5612)	Number of detected CTCs	Panel with NGS (ctDNA)	Rossi et al. [56]
Breast cancer	Immunoaffinity (EpCAM, CKs)	112 46%	5 CTCS/sample in CTC-positive patients (1–701)	Number of detected CTCs	Panel with NGS and digital droplet PCR (ESR1, PIK3CA, KRAS) (cfDNA)	Shaw et al. [57]
Follicular lymphoma	-	133 94%	7 CTCs/10^3^ peripheral blood cells in CTC-positive patients (5/10^5^–9/10^1^)	Number of detected CTCs	Digital droplet PCR (bcl2-JH) (cfDNA)	Delfau-Larue et al. [58]
Non-small cell lung cancer	Immunoaffinity (CKs)	28 33%	6.5 CTCs/sample in CTC-positive patients (1–24)	Number of detected CTCs	Real-time PCR (EGFR) (cfDNA)	Isobe et al. [59]
Non-small cell lung cancer	Microfiltration in two and three dimensions	89 91%	2 CTCs/sample in all patients (0–21)	Number of detected CTCs	Quantitative PCR (telomerase reverse transcript) (cfDNA)	Alama et al. [60]
Urothelial cancer	Immunoaffinity (EpCAM, cytokeratin)	16 75%	2.5 CTCs/sample in all patients (0–170)	Number of detected CTCs	Panel with NGS (ctDNA)	Chalfin et al. [61]
Head and neck, gastrointestinal cancer	Inertial microfluidics	37 87%	14.5 CTCs/mL in CTC-positive patients (3–133)	Genome	Panel with NGS (ctDNA)	Onidani et al. [62]
Multiple myeloma	Immunoaffinity (CD138)	28 Not provided	Not provided	Genome	Copy number alterations with WGS and WES (cfDNA)	Manier et al. [63]

CKs, cytokeratins; EpCAM, epithelial cell adhesion molecule; NGS, next-generation sequencing; PCR, polymerase chain reaction; WEG, whole-exome sequencing; WGS, whole-genome sequencing.

**Table 3:** Summary of representative circulating tumor cell (CTC) clinical trials.

Cancer Type	CTC Enrichment	Number of Patients	Objective	Treatment
Breast cancer [4]	Immunoaffinity	547	Risk stratification for late recurrence	Chemotherapy
Breast cancer [7]	Immunoaffinity	177	Predict prognosis	Chemotherapy, hormonal treatment, and immunotherapy
Pancreatic cancer [5]	Immunoaffinity	69	Predict prognosis	Surgery
Colorectal cancer [6]	Immunoaffinity	430	Predict prognosis	Chemotherapy
Prostate cancer [86]	Immunoaffinity	231	Predict prognosis	Chemotherapy

## Abbreviations

APC: adenomatous polyposis coli; cfDNA, cell-free DNA; CKs, cytokeratins; CSCs, cancer stem cells; CTCs, circulating tumor cells; ctDNA, circulating tumor DNA; DEP, dielectrophoresis; EGFR, epidermal growth factor receptor; EMT, epithelial–mesenchymal transition; EpCAM, epithelial cell adhesion molecule; iFISH, immunostaining-fluorescence in situ hybridization; LSC-MS, live single-cell mass spectrometry; NETs, neutrophil extracellular traps; NGS, next-generation sequencing; NSCLC, non-small cell lung cancer; PD-L1, programmed cell-death ligand 1; PFS, progression-free survival; PIK3CA, phosphatidylinositol-4,5-bisphosphate 3-kinase catalytic subunit alpha; WBCs, white blood cells; WEG, whole-exome sequencing; WGS, whole-genome sequencing.

## References

[1] Bray F., Ferlay J., Soerjomataram I., Siegel R.L., Torre L.A., Jemal A. (2018). Global cancer statistics 2018: GLOBOCAN estimates of incidence and mortality worldwide for 36 cancers in 185 countries. CA Cancer J. Clin..

[2] TR A. (1869). A case of cancer in which cells similar to those in the tumours were seen in the blood after death. Med. J. Aust..

[3] Pantel K., Alix-Panabieres C. (2010). Circulating tumour cells in cancer patients: Challenges and perspectives. Trends Mol. Med..

[4] Sparano J., O’Neill A., Alpaugh K., Wolff A.C., Northfelt D.W., Dang C.T., Sledge G.W., Miller K.D. (2018). Association of Circulating Tumor Cells With Late Recurrence of Estrogen Receptor-Positive Breast Cancer: A Secondary Analysis of a Randomized Clinical Trial. JAMA Oncol..

[5] Effenberger K.E., Schroeder C., Hanssen A., Wolter S., Eulenburg C., Tachezy M., Gebauer F., Izbicki J.R., Pantel K., Bockhorn M. (2018). Improved Risk Stratification by Circulating Tumor Cell Counts in Pancreatic Cancer. Clin. Cancer Res..

[6] Cohen S.J., Punt C.J., Iannotti N., Saidman B.H., Sabbath K.D., Gabrail N.Y., Picus J., Morse M., Mitchell E., Miller M.C. (2008). Relationship of circulating tumor cells to tumor response, progression-free survival, and overall survival in patients with metastatic colorectal cancer. J. Clin. Oncol..

[7] Cristofanilli M., Budd G.T., Ellis M.J., Stopeck A., Matera J., Miller M.C., Reuben J.M., Doyle G.V., Allard W.J., Terstappen L.W. (2004). Circulating tumor cells, disease progression, and survival in metastatic breast cancer. N. Engl. J. Med..

[8] Sefrioui D., Blanchard F., Toure E., Basile P., Beaussire L., Dolfus C., Perdrix A., Paresy M., Antonietti M., Iwanicki-Caron I. (2017). Diagnostic value of CA19.9, circulating tumour DNA and circulating tumour cells in patients with solid pancreatic tumours. Br. J. Cancer.

[9] Soler A., Cayrefourcq L., Mazard T., Babayan A., Lamy P.J., Assou S., Assenat E., Pantel K., Alix-Panabieres C. (2018). Autologous cell lines from circulating colon cancer cells captured from sequential liquid biopsies as model to study therapy-driven tumor changes. Sci. Rep..

[10] Giannopoulou L., Kasimir-Bauer S., Lianidou E.S. (2018). Liquid biopsy in ovarian cancer: Recent advances on circulating tumor cells and circulating tumor DNA. Clin. Chem. Lab. Med..

[11] Rhim A.D., Mirek E.T., Aiello N.M., Maitra A., Bailey J.M., McAllister F., Reichert M., Beatty G.L., Rustgi A.K., Vonderheide R.H. (2012). EMT and dissemination precede pancreatic tumor formation. Cell.

[12] Allard W.J., Matera J., Miller M.C., Repollet M., Connelly M.C., Rao C., Tibbe A.G., Uhr J.W., Terstappen L.W. (2004). Tumor cells circulate in the peripheral blood of all major carcinomas but not in healthy subjects or patients with nonmalignant diseases. Clin. Cancer Res..

[13] Baccelli I., Schneeweiss A., Riethdorf S., Stenzinger A., Schillert A., Vogel V., Klein C., Saini M., Bauerle T., Wallwiener M. (2013). Identification of a population of blood circulating tumor cells from breast cancer patients that initiates metastasis in a xenograft assay. Nat. Biotechnol..

[14] Venugopal Menon N., Lim S.B., Lim C.T. (2019). Microfluidics for personalized drug screening of cancer. Curr. Opin. Pharmacol..

[15] Dagogo-Jack I., Shaw A.T. (2018). Tumour heterogeneity and resistance to cancer therapies. Nat. Rev. Clin. Oncol..

[16] Jamal-Hanjani M., Wilson G.A., McGranahan N., Birkbak N.J., Watkins T.B.K., Veeriah S., Shafi S., Johnson D.H., Mitter R., Rosenthal R. (2017). Tracking the Evolution of Non-Small-Cell Lung Cancer. N. Engl. J. Med..

[17] Gerlinger M., Horswell S., Larkin J., Rowan A.J., Salm M.P., Varela I., Fisher R., McGranahan N., Matthews N., Santos C.R. (2014). Genomic architecture and evolution of clear cell renal cell carcinomas defined by multiregion sequencing. Nat. Genet..

[18] Harbst K., Lauss M., Cirenajwis H., Isaksson K., Rosengren F., Torngren T., Kvist A., Johansson M.C., Vallon-Christersson J., Baldetorp B. (2016). Multiregion Whole-Exome Sequencing Uncovers the Genetic Evolution and Mutational Heterogeneity of Early-Stage Metastatic Melanoma. Cancer Res..

[19] Yates L.R., Gerstung M., Knappskog S., Desmedt C., Gundem G., Van Loo P., Aas T., Alexandrov L.B., Larsimont D., Davies H. (2015). Subclonal diversification of primary breast cancer revealed by multiregion sequencing. Nat. Med..

[20] Johnson B.E., Mazor T., Hong C., Barnes M., Aihara K., McLean C.Y., Fouse S.D., Yamamoto S., Ueda H., Tatsuno K. (2014). Mutational analysis reveals the origin and therapy-driven evolution of recurrent glioma. Science.

[21] Mitsudomi T., Suda K., Yatabe Y. (2013). Surgery for NSCLC in the era of personalized medicine. Nat. Rev. Clin. Oncol..

[22] Heitzer E., Auer M., Gasch C., Pichler M., Ulz P., Hoffmann E.M., Lax S., Waldispuehl-Geigl J., Mauermann O., Lackner C. (2013). Complex tumor genomes inferred from single circulating tumor cells by array-CGH and next-generation sequencing. Cancer Res..

[23] Ledford H. (2011). Cancer theory faces doubts. Nature.

[24] Pantel K., Brakenhoff R.H. (2004). Dissecting the metastatic cascade. Nat. Rev. Cancer.

[25] Ksiazkiewicz M., Markiewicz A., Zaczek A.J. (2012). Epithelial-mesenchymal transition: A hallmark in metastasis formation linking circulating tumor cells and cancer stem cells. Pathobiology.

[26] Qi L.N., Xiang B.D., Wu F.X., Ye J.Z., Zhong J.H., Wang Y.Y., Chen Y.Y., Chen Z.S., Ma L., Chen J. (2018). Circulating Tumor Cells Undergoing EMT Provide a Metric for Diagnosis and Prognosis of Patients with Hepatocellular Carcinoma. Cancer Res..

[27] Agnoletto C., Corra F., Minotti L., Baldassari F., Crudele F., Cook W.J.J., Di Leva G., d’Adamo A.P., Gasparini P., Volinia S. (2019). Heterogeneity in Circulating Tumor Cells: The Relevance of the Stem-Cell Subset. Cancers (Basel).

[28] Al-Hajj M., Wicha M.S., Benito-Hernandez A., Morrison S.J., Clarke M.F. (2003). Prospective identification of tumorigenic breast cancer cells. Proc. Natl. Acad. Sci. USA.

[29] Liu R., Wang X., Chen G.Y., Dalerba P., Gurney A., Hoey T., Sherlock G., Lewicki J., Shedden K., Clarke M.F. (2007). The prognostic role of a gene signature from tumorigenic breast-cancer cells. N. Engl. J. Med..

[30] Ginestier C., Hur M.H., Charafe-Jauffret E., Monville F., Dutcher J., Brown M., Jacquemier J., Viens P., Kleer C.G., Liu S. (2007). ALDH1 is a marker of normal and malignant human mammary stem cells and a predictor of poor clinical outcome. Cell Stem Cell.

[31] Mani S.A., Guo W., Liao M.J., Eaton E.N., Ayyanan A., Zhou A.Y., Brooks M., Reinhard F., Zhang C.C., Shipitsin M. (2008). The epithelial-mesenchymal transition generates cells with properties of stem cells. Cell.

[32] Morel A.P., Lievre M., Thomas C., Hinkal G., Ansieau S., Puisieux A. (2008). Generation of breast cancer stem cells through epithelial-mesenchymal transition. PLoS ONE.

[33] Ebright R.Y., Lee S., Wittner B.S., Niederhoffer K.L., Nicholson B.T., Bardia A., Truesdell S., Wiley D.F., Wesley B., Li S. (2020). Deregulation of ribosomal protein expression and translation promotes breast cancer metastasis. Science.

[34] Liu X., Li J., Cadilha B.L., Markota A., Voigt C., Huang Z., Lin P.P., Wang D.D., Dai J., Kranz G. (2019). Epithelial-type systemic breast carcinoma cells with a restricted mesenchymal transition are a major source of metastasis. Sci. Adv..

[35] Lim S.B., Di Lee W., Vasudevan J., Lim W.T., Lim C.T. (2019). Liquid biopsy: One cell at a time. NPJ Precis. Oncol..

[36] Gabriel M.T., Calleja L.R., Chalopin A., Ory B., Heymann D. (2016). Circulating Tumor Cells: A Review of Non-EpCAM-Based Approaches for Cell Enrichment and Isolation. Clin. Chem..

[37] Steinert G., Scholch S., Niemietz T., Iwata N., Garcia S.A., Behrens B., Voigt A., Kloor M., Benner A., Bork U. (2014). Immune escape and survival mechanisms in circulating tumor cells of colorectal cancer. Cancer Res..

[38] Blassl C., Kuhlmann J.D., Webers A., Wimberger P., Fehm T., Neubauer H. (2016). Gene expression profiling of single circulating tumor cells in ovarian cancer—Establishment of a multi-marker gene panel. Mol. Oncol..

[39] Hao S.J., Wan Y., Xia Y.Q., Zou X., Zheng S.Y. (2018). Size-based separation methods of circulating tumor cells. Adv. Drug Deliv. Rev..

[40] Gorges T.M., Kuske A., Rock K., Mauermann O., Muller V., Peine S., Verpoort K., Novosadova V., Kubista M., Riethdorf S. (2016). Accession of Tumor Heterogeneity by Multiplex Transcriptome Profiling of Single Circulating Tumor Cells. Clin. Chem..

[41] Chen C.L., Mahalingam D., Osmulski P., Jadhav R.R., Wang C.M., Leach R.J., Chang T.C., Weitman S.D., Kumar A.P., Sun L. (2013). Single-cell analysis of circulating tumor cells identifies cumulative expression patterns of EMT-related genes in metastatic prostate cancer. Prostate.

[42] Ferreira M.M., Ramani V.C., Jeffrey S.S. (2016). Circulating tumor cell technologies. Mol. Oncol..

[43] Hou H.W., Warkiani M.E., Khoo B.L., Li Z.R., Soo R.A., Tan D.S., Lim W.T., Han J., Bhagat A.A., Lim C.T. (2013). Isolation and retrieval of circulating tumor cells using centrifugal forces. Sci. Rep..

[44] Meye A., Bilkenroth U., Schmidt U., Füssel S., Robel K., Melchior A.M., Blümke K., Pinkert D., Bartel F., Linne C. (2002). Isolation and enrichment of urologic tumor cells in blood samples by a semi-automated CD45 depletion autoMACS protocol. Int. J. Oncol..

[45] Lohr J.G., Kim S., Gould J., Knoechel B., Drier Y., Cotton M.J., Gray D., Birrer N., Wong B., Ha G. (2016). Genetic interrogation of circulating multiple myeloma cells at single-cell resolution. Sci. Transl. Med..

[46] Ramskold D., Luo S., Wang Y.C., Li R., Deng Q., Faridani O.R., Daniels G.A., Khrebtukova I., Loring J.F., Laurent L.C. (2012). Full-length mRNA-Seq from single-cell levels of RNA and individual circulating tumor cells. Nat. Biotechnol..

[47] Powell A.A., Talasaz A.H., Zhang H., Coram M.A., Reddy A., Deng G., Telli M.L., Advani R.H., Carlson R.W., Mollick J.A. (2012). Single cell profiling of circulating tumor cells: Transcriptional heterogeneity and diversity from breast cancer cell lines. PLoS ONE.

[48] Park S.M., Wong D.J., Ooi C.C., Kurtz D.M., Vermesh O., Aalipour A., Suh S., Pian K.L., Chabon J.J., Lee S.H. (2016). Molecular profiling of single circulating tumor cells from lung cancer patients. Proc. Natl. Acad. Sci. USA.

[49] Cann G.M., Gulzar Z.G., Cooper S., Li R., Luo S., Tat M., Stuart S., Schroth G., Srinivas S., Ronaghi M. (2012). mRNA-Seq of single prostate cancer circulating tumor cells reveals recapitulation of gene expression and pathways found in prostate cancer. PLoS ONE.

[50] Li Y., Zhang X., Ge S., Gao J., Gong J., Lu M., Zhang Q., Cao Y., Wang D.D., Lin P.P. (2014). Clinical significance of phenotyping and karyotyping of circulating tumor cells in patients with advanced gastric cancer. Oncotarget.

[51] Pantel K., Deneve E., Nocca D., Coffy A., Vendrell J.P., Maudelonde T., Riethdorf S., Alix-Panabières C. (2012). Circulating epithelial cells in patients with benign colon diseases. Clin. Chem..

[52] Che J., Yu V., Dhar M., Renier C., Matsumoto M., Heirich K., Garon E.B., Goldman J., Rao J., Sledge G.W. (2016). Classification of large circulating tumor cells isolated with ultra-high throughput microfluidic Vortex technology. Oncotarget.

[53] Hvichia G.E., Parveen Z., Wagner C., Janning M., Quidde J., Stein A., Müller V., Loges S., Neves R.P., Stoecklein N.H. (2016). A novel microfluidic platform for size and deformability based separation and the subsequent molecular characterization of viable circulating tumor cells. Int. J. Cancer.

[54] Wong V.C., Ko J.M., Lam C.T., Lung M.L. (2017). Succinct workflows for circulating tumor cells after enrichment: From systematic counting to mutational profiling. PLoS ONE.

[55] Lee Y., Guan G., Bhagat A.A. (2018). ClearCell(R) FX, a label-free microfluidics technology for enrichment of viable circulating tumor cells. Cytom. A.

[56] Rossi G., Mu Z., Rademaker A.W., Austin L.K., Strickland K.S., Costa R.L.B., Nagy R.J., Zagonel V., Taxter T.J., Behdad A. (2018). Cell-Free DNA and Circulating Tumor Cells: Comprehensive Liquid Biopsy Analysis in Advanced Breast Cancer. Clin. Cancer Res..

[57] Shaw J.A., Guttery D.S., Hills A., Fernandez-Garcia D., Page K., Rosales B.M., Goddard K.S., Hastings R.K., Luo J., Ogle O. (2017). Mutation Analysis of Cell-Free DNA and Single Circulating Tumor Cells in Metastatic Breast Cancer Patients with High Circulating Tumor Cell Counts. Clin. Cancer Res..

[58] Delfau-Larue M.H., van der Gucht A., Dupuis J., Jais J.P., Nel I., Beldi-Ferchiou A., Hamdane S., Benmaad I., Laboure G., Verret B. (2018). Total metabolic tumor volume, circulating tumor cells, cell-free DNA: Distinct prognostic value in follicular lymphoma. Blood Adv..

[59] Isobe K., Hata Y., Kobayashi K., Hirota N., Sato K., Sano G., Sugino K., Sakamoto S., Takai Y., Shibuya K. (2012). Clinical significance of circulating tumor cells and free DNA in non-small cell lung cancer. Anticancer Res..

[60] Alama A., Coco S., Genova C., Rossi G., Fontana V., Tagliamento M., Giovanna Dal Bello M., Rosa A., Boccardo S., Rijavec E. (2019). Prognostic Relevance of Circulating Tumor Cells and Circulating Cell-Free DNA Association in Metastatic Non-Small Cell Lung Cancer Treated with Nivolumab. J. Clin. Med..

[61] Chalfin H.J., Glavaris S.A., Gorin M.A., Kates M.R., Fong M.H., Dong L., Matoso A., Bivalacqua T.J., Johnson M.H., Pienta K.J. (2019). Circulating Tumor Cell and Circulating Tumor DNA Assays Reveal Complementary Information for Patients with Metastatic Urothelial Cancer. Eur. Urol. Oncol..

[62] Onidani K., Shoji H., Kakizaki T., Yoshimoto S., Okaya S., Miura N., Sekikawa S., Furuta K., Lim C.T., Shibahara T. (2019). Monitoring of cancer patients via next-generation sequencing of patient-derived circulating tumor cells and tumor DNA. Cancer Sci..

[63] Manier S., Park J., Capelletti M., Bustoros M., Freeman S.S., Ha G., Rhoades J., Liu C.J., Huynh D., Reed S.C. (2018). Whole-exome sequencing of cell-free DNA and circulating tumor cells in multiple myeloma. Nat. Commun..

[64] Abouleila Y., Onidani K., Ali A., Shoji H., Kawai T., Lim C.T., Kumar V., Okaya S., Kato K., Hiyama E. (2019). Live single cell mass spectrometry reveals cancer-specific metabolic profiles of circulating tumor cells. Cancer Sci..

[65] Kim H.Y., Lee K.M., Kim S.H., Kwon Y.J., Chun Y.J., Choi H.K. (2016). Comparative metabolic and lipidomic profiling of human breast cancer cells with different metastatic potentials. Oncotarget.

[66] Boroughs L.K., DeBerardinis R.J. (2015). Metabolic pathways promoting cancer cell survival and growth. Nat. Cell Biol..

[67] Brown D.G., Rao S., Weir T.L., O’Malia J., Bazan M., Brown R.J., Ryan E.P. (2016). Metabolomics and metabolic pathway networks from human colorectal cancers, adjacent mucosa, and stool. Cancer Metab..

[68] Hiyama E., Ali A., Amer S., Harada T., Shimamoto K., Furushima R., Abouleila Y., Emara S., Masujima T. (2015). Direct Lipido-Metabolomics of Single Floating Cells for Analysis of Circulating Tumor Cells by Live Single-cell Mass Spectrometry. Anal. Sci..

[69] Fujii T., Matsuda S., Tejedor M.L., Esaki T., Sakane I., Mizuno H., Tsuyama N., Masujima T. (2015). Direct metabolomics for plant cells by live single-cell mass spectrometry. Nat. Protoc..

[70] Xu J., Chen Y., Zhang R., He J., Song Y., Wang J., Wang H., Wang L., Zhan Q., Abliz Z. (2016). Global metabolomics reveals potential urinary biomarkers of esophageal squamous cell carcinoma for diagnosis and staging. Sci. Rep..

[71] Stott S.L., Hsu C.H., Tsukrov D.I., Yu M., Miyamoto D.T., Waltman B.A., Rothenberg S.M., Shah A.M., Smas M.E., Korir G.K. (2010). Isolation of circulating tumor cells using a microvortex-generating herringbone-chip. Proc. Natl. Acad. Sci. USA.

[72] Sarioglu A.F., Aceto N., Kojic N., Donaldson M.C., Zeinali M., Hamza B., Engstrom A., Zhu H., Sundaresan T.K., Miyamoto D.T. (2015). A microfluidic device for label-free, physical capture of circulating tumor cell clusters. Nat. Methods.

[73] Saini M., Szczerba B.M., Aceto N. (2019). Circulating Tumor Cell-Neutrophil Tango along the Metastatic Process. Cancer Res..

[74] Aceto N., Bardia A., Miyamoto D.T., Donaldson M.C., Wittner B.S., Spencer J.A., Yu M., Pely A., Engstrom A., Zhu H. (2014). Circulating tumor cell clusters are oligoclonal precursors of breast cancer metastasis. Cell.

[75] Wang C., Mu Z., Chervoneva I., Austin L., Ye Z., Rossi G., Palazzo J.P., Sun C., Abu-Khalaf M., Myers R.E. (2017). Longitudinally collected CTCs and CTC-clusters and clinical outcomes of metastatic breast cancer. Breast Cancer Res. Treat..

[76] Szczerba B.M., Castro-Giner F., Vetter M., Krol I., Gkountela S., Landin J., Scheidmann M.C., Donato C., Scherrer R., Singer J. (2019). Neutrophils escort circulating tumour cells to enable cell cycle progression. Nature.

[77] Cools-Lartigue J., Spicer J., McDonald B., Gowing S., Chow S., Giannias B., Bourdeau F., Kubes P., Ferri L. (2013). Neutrophil extracellular traps sequester circulating tumor cells and promote metastasis. J. Clin. Investig..

[78] Liao C.J., Hsieh C.H., Hung F.C., Wang H.M., Chou W.P., Wu M.H. (2019). The Integration of a Three-Dimensional Spheroid Cell Culture Operation in a Circulating Tumor Cell (CTC) Isolation and Purification Process: A Preliminary Study of the Clinical Significance and Prognostic Role of the CTCs Isolated from the Blood Samples of Head and Neck Cancer Patients. Cancers (Basel).

[79] Zhao P., Zhou W., Liu C., Zhang H., Cheng Z., Wu W., Liu K., Hu H., Zhong C., Zhang Y. (2019). Establishment and Characterization of a CTC Cell Line from Peripheral Blood of Breast Cancer Patient. J. Cancer.

[80] De T., Goyal S., Balachander G., Chatterjee K., Kumar P., Babu K.G., Rangarajan A. (2019). A Novel Ex Vivo System Using 3D Polymer Scaffold to Culture Circulating Tumor Cells from Breast Cancer Patients Exhibits Dynamic E-M Phenotypes. J. Clin. Med..

[81] Khoo B.L., Grenci G., Jing T., Lim Y.B., Lee S.C., Thiery J.P., Han J., Lim C.T. (2016). Liquid biopsy and therapeutic response: Circulating tumor cell cultures for evaluation of anticancer treatment. Sci. Adv..

[82] Yachida S., Jones S., Bozic I., Antal T., Leary R., Fu B., Kamiyama M., Hruban R.H., Eshleman J.R., Nowak M.A. (2010). Distant metastasis occurs late during the genetic evolution of pancreatic cancer. Nature.

[83] Keller L., Pantel K. (2019). Unravelling tumour heterogeneity by single-cell profiling of circulating tumour cells. Nat. Rev. Cancer.

[84] Khattak M.A., Reid A., Freeman J., Pereira M., McEvoy A., Lo J., Frank M.H., Meniawy T., Didan A., Spencer I. (2019). PD-L1 Expression on Circulating Tumor Cells May Be Predictive of Response to Pembrolizumab in Advanced Melanoma: Results from a Pilot Study. Oncologist.

[85] Janning M., Kobus F., Babayan A., Wikman H., Velthaus J.L., Bergmann S., Schatz S., Falk M., Berger L.A., Bottcher L.M. (2019). Determination of PD-L1 Expression in Circulating Tumor Cells of NSCLC Patients and Correlation with Response to PD-1/PD-L1 Inhibitors. Cancers (Basel).

[86] De Bono J.S., Scher H.I., Montgomery R.B., Parker C., Miller M.C., Tissing H., Doyle G.V., Terstappen L.W., Pienta K.J., Raghavan D. (2008). Circulating tumor cells predict survival benefit from treatment in metastatic castration-resistant prostate cancer. Clin. Cancer Res..

[87] Satelli A., Li S. (2011). Vimentin in cancer and its potential as a molecular target for cancer therapy. Cell. Mol. Life Sci..

